# New walking and cycling infrastructure and modal shift in the UK: A quasi-experimental panel study

**DOI:** 10.1016/j.tra.2016.11.017

**Published:** 2017-01

**Authors:** Yena Song, John Preston, David Ogilvie, on behalf of the iConnect consortium

**Affiliations:** a Department of Geography, Chonnam National University, Gwangju 61186, South Korea; b Transportation Research Group, Faculty of Engineering and the Environment, University of Southampton, Building 176, Bodlrewood, Southampton SO16 7QF, UK; c Medical Research Council Epidemiology Unit and UKCRC Centre for Diet and Activity Research (CEDAR), University of Cambridge School of Clinical Medicine, Box 285, Cambridge Biomedical Campus, Cambridge CB2 0QQ, UK

**Keywords:** Walking and cycling, Active travel, Modal shift, Infrastructure intervention, Travel behaviour, Evaluation

## Abstract

Heavy dependency on car use leads to traffic congestion, pollution, and physical inactivity, which impose high direct and indirect costs on society. Promoting walking and cycling has been recognised as one of the means of mitigating such negative effects. Various approaches have been taken to enhance walking and cycling levels and to reduce the use of automobiles. This paper examines the effectiveness of infrastructure interventions in promoting walking and cycling for transport. Two related sets of panel data, covering elapsed time periods of one and two years, were analysed to track changes in travel behaviour following provision of new walking and cycling infrastructure so that modal shift from private car use to walking and cycling can be investigated. Two types of exposure measures were tested: distance from the infrastructure (a measure of potential usage), and actual usage of the infrastructure. Only the latter measure was statistically significantly associated with modal shift. This in turn suggested that infrastructure provision was not a sufficient condition for modal shift, but may have been a necessary condition. Along with the use of new infrastructure, the loss of employment, higher education, being male and being part of the ethnic majority were consistently found to be significantly and positively associated with modal shift towards walking and cycling. The findings of this study support the construction of walking and cycling routes, but also suggest that such infrastructure alone may not be enough to promote active travel.

## 1 Introduction

Increasing auto dependency has resulted in serious environmental and societal repercussions, but such negative effects can be alleviated by reducing car use and stimulating the use of more environmentally friendly transport modes (Cools et al., 2009, Marshall and Banister, 2000). A modal shift towards active travel modes such as walking and cycling has various potential positive impacts. It could reduce air pollution from burning fossil fuels, mitigate traffic congestion, increase levels of physical activity and lead to more sustainable communities (Banister, 2008, Rissel, 2009, Giles-Corti et al., 2010).

In order to suppress car use and, at the same time, to promote active travel, various transport policies and strategies have been implemented through a wide range of structural, infrastructural or behavioural interventions (Graham-Rowe et al., 2011). Many studies have found a positive association between such transport interventions and active travel (Brownson et al., 2000, Moudon et al., 2005, Mutrie et al., 2002, Ogilvie et al., 2007, Parkin et al., 2008, Pucher et al., 2011, Rietveld and Daniel, 2004, Wen et al., 2005, Yang et al., 2010). Furthermore, recent cost-benefit studies of walking and cycling infrastructure report high benefit-cost ratios, implying that construction of such infrastructure can be beneficial to society (Cavill et al., 2008, Davids, 2010, Gotschl, 2011, Sælensminde, 2004, Wang et al., 2005). However, most empirical studies in this field have been based on cross-sectional data and/or did not include control groups in their study design, which limits their capacity to support causal inference about the effectiveness of the interventions (Powell et al., 2010). Therefore, although there has been much research on active travel and infrastructural interventions, more rigorous longitudinal studies are needed (Graham-Rowe et al., 2011, Krizek et al., 2009). A panel study offers a useful way of evaluating a transport intervention and of investigating dynamic aspects of travel behaviour (Kitamura, 1990).

In the last few years, substantial investments in walking and cycling infrastructure have been made across the UK (Davids, 2010, Powell et al., 2010, Redfern et al., 2011). This study aims to contribute to the empirical evidence base by exploring and analysing panel data obtained in a quasi-experimental study of three selected sites of a national programme of constructing new walking and cycling routes. In this paper we evaluate the effectiveness of this infrastructure in promoting a modal shift. More specifically, we examine the travel behaviour change that can be stimulated by infrastructural interventions to improve walking and cycling facilities by addressing two research questions: (1) Does exposure to transport infrastructure interventions encourage a modal shift towards walking and cycling? and (2) Which groups of people are more likely to incorporate active travel in their journeys once such an intervention is implemented?

## 2 Methods

### 2.1 Interventions

We used the data collected as part of the iConnect (Impact of Constructing Non-motorised Networks and Evaluating Changes in Travel) study, which aimed to evaluate the impact of a walking and cycling infrastructure programme called Connect2. This programme initially comprised 84 transport infrastructure projects across the UK that built and/or improved walking and cycling routes and thereby aimed to promote active travel in the general population. Of the initial 84 sites, three – in Cardiff, Kenilworth and Southampton – were selected for the core evaluation study after considering the heterogeneity of local contexts, likely impacts, construction timetables and accessibility to researchers of the available sites (Ogilvie et al., 2012).

**Fig. 1:**
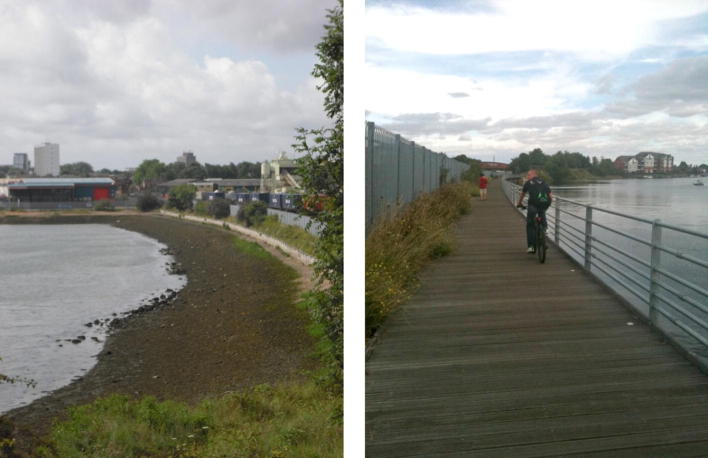
Pre- and post-intervention conditions in Southampton. Photographs by Yena Song (left: before construction, July 2009, right: after construction, August 2011).

In the three study sites, new walking and cycling infrastructure has been constructed and existing routes have been improved. Connect2 in Cardiff includes construction of a traffic-free pedestrian/cyclist bridge over the River Ely, called The Peoples’ Bridge, connecting the suburb of Penarth to the city centre and involving development of feeder routes to and from the bridge. Southampton’s intervention involved building a raised boardwalk linking the city centre and nearby residential areas along the shore of the River Itchen, where an informal footpath and feeder routes had existed before (Fig. 1). The core element of Kenilworth’s intervention was a walking and cycling bridge crossing a busy dual carriageway. Other elements in Kenilworth entailed improving the existing greenway between Balsall Common and Kenilworth town centre and building a cycle route between Warwick University and Kenilworth town centre. The Peoples’ Bridge in Cardiff and the boardwalk in Southampton have been in use since July 2010. The core element of the Kenilworth scheme was implemented in September 2011, but the link between Kenilworth and the University was implemented after the iConnect data collection was completed.

### 2.2 Study design

We collected travel behaviour data as well as personal and household information during late spring/early summer in 2010, 2011 and 2012 from the populations living around the case study sites. The aim was to study changes in travel behaviour before and after the construction of the new walking and cycling infrastructure.

7500 adults living within 5 km by road of the core of each intervention site were randomly selected from the edited electoral register and mailed a baseline survey package including a questionnaire, consent form and participant information sheet in April 2010^1^. The questionnaire collected data on personal and household characteristics and the weekly travel and physical activity of each respondent, the latter being shown to have comparable reliability and validity with that of more established postal survey instruments (Adams et al., 2014).

In total, 3516 people returned completed or partially completed baseline questionnaires, which made for a 15.6% response rate. At the same time of year in 2011 and 2012, all the baseline survey respondents were sent follow-up surveys that asked the same questions along with one additional section related to their awareness and use of the new infrastructure in their local area. In the 2011 follow-up, 1906 completed questionnaires were returned, a 54.2% retention rate. In 2012, 1564 people responded to the second follow-up survey, a 44.5% retention rate from baseline.

### 2.3 Study data

The sample responding to the baseline survey differed somewhat from the local or national population (Sahlqvist et al., 2012, Song et al., 2013). Our respondents were more likely to be female, older, and white, to have higher household car access, and to have achieved a higher level of education than the general population. In the samples retained in the two follow-up surveys, these response patterns were sustained or strengthened. Table 1 reports the demographic and household characteristics of the samples responding to the three waves of the survey. The proportions of younger people, ethnic minorities, households owning multiple cars, paid workers and students decreased and the average age of the sample increased over time. This implies a degree of selective attrition in the longitudinal survey responses, although the decreased proportions of working and student groups would partly reflect the ageing of the sample and the declining number of household cars corresponds with the general trend^2^.

In this study we used the data of those who responded to either of the first or second follow-up surveys. As these were mailed only to the baseline survey respondents, we were able to build two panel data sets, i.e. those including 2010–2011 and 2010–2012 data, and to investigate travel behaviour changes over the one and two years following baseline data collection. In the analysis we excluded those who had moved home between the two waves of the survey, whose responses at different time points appeared to refer to different persons, or who did not provide travel information at either baseline or follow-up. Also, to mitigate the limitations inherent in the representativeness and selective attrition of the samples, a calibration weight was developed and applied for each panel data set. The calibration weights adjusted the age and gender distribution of the achieved samples to reflect the local population demographics in the 2011 Census. The resultant sample sizes used for the following analysis were 1829 and 1489 for 2010–2011 and 2010–2012 respectively.

## 3 Travel behaviour change

### 3.1 Aggregate description of travel behaviour change

The survey recipients were asked to provide a summary of their travel activity in the previous seven days in terms of the total travel time, and total distance travelled, by seven pre-specified modal categories: walking, cycling, bus, train, car as a driver, car as a passenger and other. By travel we meant only those journeys made for utility purposes such as commuting, business, shopping, healthcare or social activities. Walking or cycling without an utility purpose of moving from place to place, e.g. strolling, dog walking or recreational cycling, were not included in this analysis, but participants were asked to record these behaviours in a separate, physical activity, section of the questionnaire and they were analysed separately (Goodman et al., 2014).

**Fig. 2:**
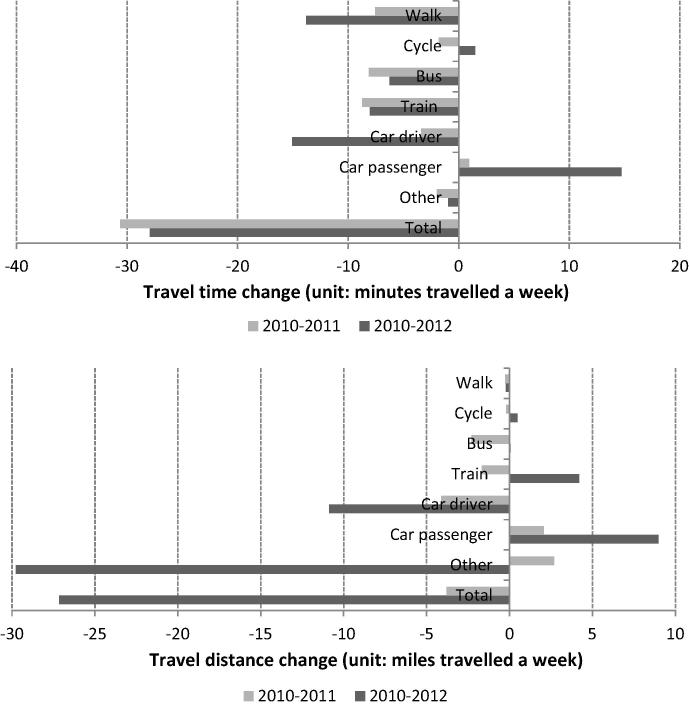
Changes in mean weekly travel time (minutes) and distance (miles) by mode.

Fig. 2 presents the differences in the sample means summarising the use of the different travel modes between the baseline and the two follow-up surveys. The mean values for 2010 were computed only for those who took part in the relevant follow-up. The mean absolute weekly quantity of travel decreased over time for most modes. A relatively large decrease in travel distance was observed for the use of ‘other’ modes, which mostly reflects a reduction in air travel. Journeys by air tend to increase the average travel distance significantly. For instance, one additional return journey between New York City and London made by a single respondent could increase the average weekly travel distance of the whole sample by 3.8 miles for the 2010–2011 panel and by 4.6 miles for 2010–2012 panel. Disregarding flights, mean weekly travel time and distance by ‘other’ modes increased slightly (by 2.17 min and 1.48 miles respectively) in 2012, having shown a decrease of 2.40 min and an increase of 0.56 miles respectively in 2011. Excluding the use of ‘other’ modes, the largest changes were noted in car journeys, with a decrease in driving and an increase in car travel as a passenger indicating that people were more likely to travel with others over time, partly reflecting a fall in the mean number of cars available to the household (Table 1). This might be expected to lead to a lower number of single-occupant vehicles on the road. Both travel time and distance cycled increased on average between 2010 and 2012, but not between 2010 and 2011.

To further compare each individual’s weekly travel time and distance between baseline and follow-up, we conducted a series of paired sample t-tests for the various travel modes separately and in combination (Table 2). The p-values in Table 2 indicate the statistical significance of each test, i.e. a value of 0.05 means that there is a 5% chance of observing a change of that magnitude between baseline and follow-up by chance. As such, lower p-values indicate a lower likelihood that differences were due to random variation and a greater likelihood that they were real. The *t*-test results revealed statistically significant decreases in the total quantity of travel between the baseline (2010) and both follow-up surveys (2011 and 2012). Time spent travelling by public transport, i.e. bus or train, decreased significantly between 2010 and 2011. After an additional year these changes remained significant, and changes in car use and walking time also became significant.

A commonly used metric to measure a mode switch or modal shift is the modal split (Marshall and Banister, 2000, National Research Center, 2010). This allows us to study travel behaviour controlling for differences in individuals’ travel demand and constraints (Song et al., 2013) as well as changes in their circumstances. Table 3 shows modal split computed in two ways: first as the share of each individual’s total travel *time* accounted for by active travel and by car driving, and second as the corresponding shares of total travel *distance*, because the Connect2 interventions were intended to induce a modal shift away from private car use towards walking and cycling (Sahlqvist et al., 2015).

Overall, after controlling for the quantity of travel, people’s journeys involved marginally more active travel and less car driving at follow-up than they did at baseline. However, variation was noticed between the sites. Southampton showed the largest changes in the intended direction. Kenilworth also showed a modal shift towards active travel, although not as much as in Southampton. In Cardiff, by contrast, the active mode share decreased and the driving mode share increased.

### 3.2 Modal shift and new walking and cycling infrastructure

The walking and cycling infrastructure built as part of the Connect2 programme was intended to enhance the quality of active travel environments and thereby to lead people to incorporate more active travel into their daily journeys, which would result in a modal shift from private motor vehicles to walking and cycling. To investigate the extent to which any travel behaviour change was attributable to the new infrastructure, we first defined those individuals whose modal split for active modes increased, and whose modal split for car driving decreased, as having ‘shifted’ their mode of travel as intended.

**Fig. 3:**
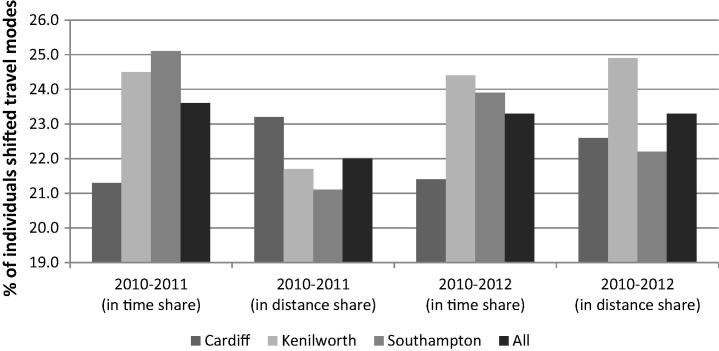
Percentages of respondents who shifted from driving to walking and cycling, by site.

Fig. 3 presents the proportions of individuals whose personal modal split shifted from driving to active modes over the study periods. About 21–25% of respondents were identified as having made such a shift, with modest variation between sites. On the other hand, the inverse shift (i.e. towards more car driving and less walking and cycling) was observed as well, with approximately 23% and 20% of respondents exhibiting this shift in terms of travel time and distance respectively in both follow-up years.

Although over 20% of respondents shifted from car driving towards active travel, given that a similar proportion exhibited an inverse shift, we cannot necessarily conclude that these changes reflected anything other than random variation or were causally related to the provision of the new infrastructure. We therefore developed statistical models to investigate the independent contributions to modal shift of five sets of explanatory variables collected in the questionnaires: (1) time-invariant personal characteristics; (2) changes in socioeconomic status; (3) changes in access to private vehicles; (4) exposure to the new walking and cycling infrastructure; and (5) the completeness of that infrastructure.

We hypothesised that those who were more exposed, or had greater accessibility, to the infrastructure would be more likely to shift from car driving towards active travel. Two measures of exposure were defined in terms of proximity, one to ‘Core C2′ and the other to ‘Greater C2′. All Connect2 projects have a landmark core engineering project such as the construction of a footbridge, walkway and so on, and Core C2 refers to this element within each Connect2 project. On the other hand, Greater C2 includes the feeder road and path networks that connect to the core elements of the new infrastructure, and was defined using information provided by key stakeholders involved in each Connect2 project. Self-reported use of the new infrastructure was employed as a third exposure measure.

Table 4 summarises the explanatory variables included in the modelling process. Missing values were imputed using the multiple imputation method, assuming that omissions occurred randomly and Rubin’s rule was applied in the estimation process (Rubin, 1987).

As the dependent variable was a dummy indicating modal shift^3^, a binary logit model (Eq. (1)) was employed in model estimation.

(1) $$\text{Modal shift from car driving towards walking and cycling}=f(X)=\frac{1}{1+e^{-\beta X}}$$

Then $\ln\frac{f(X)}{1-f(X)}=\beta X=\beta_{0}+\beta_{1}x_{1}+\beta_{2}x_{2}+\beta_{3}x_{3}+\beta_{4}x_{4}+\beta_{5}x_{5}+\beta_{6}x_{6}+\beta_{7}x_{7}+\beta_{8}x_{8}+\beta_{9}x_{9}+\beta_{10}x_{10}+\beta_{11}x_{11}$, which is equivalent to $\frac{f(X)}{1-f(X)}=e^{\beta X}$

where *X* is a vector of selected explanatory variables and *β* is a vector of estimated coefficients. More specifically, x_1_ indicates job loss dummy, x_2_ new job dummy, x_3_ change in family size, x_4_ and x_5_ changes in household bicycles and cars respectively, x_6_ age, x_7_ gender, x_8_ ethnic minority, x_9_ higher education, x_10_ intervention exposure and x_11_ infrastructure completed.

Table 5, Table 6 present the model outputs for the 2010–2011 and 2010–2012 changes respectively. All models were found to be statistically significant, in that the *F*-values were high enough to conclude that the explanatory variables included in those models did significantly predict the modal shift, with there being in all cases a less than 10% chance of no modelled effect, and a less than 1% chance in most cases. However, the two-year models tended to have higher *F*-values than the one-year models, and those based on changes in travel time were better modelled than those based on changes in distance. This may reflect the time taken to change travel behaviour, or a combination of the greater measurement error and prevalence of missing values for travel distance.

Only one exposure measure was used for each model, to avoid redundancy and to identify the exposure measure(s) most strongly related to behaviour change. The coefficients for all exposure variables had the expected signs, implying a positive association of modal shift with both proximity to, and actual use of, the intervention. However, the only exposure measure found to be significantly associated was that representing use of the new or upgraded infrastructure^4^. This suggests that while actual users were more likely to show the intended travel behaviour change than non-users, living closer to the new infrastructure was not in itself significantly associated with this outcome, which undermines the case for causal inference linking the provision of the new infrastructure with travel behaviour change.

Losing a job was identified as significantly increasing the chance of a modal shift in all models, although acquiring a new job was not associated with this outcome. Level of education was consistently and positively associated in all modelling combinations, indicating that those with higher education were more likely to make a modal shift, while women were less likely to do so in all models. An increase in family size was positively associated with modal shift in the first year, but the sign of this association changed in the second year of follow-up. Being a member of an ethnic minority was negatively associated with modal shift in most models except for the one-year travel distance model. A change in household access to vehicles was strongly associated with modal shift in the two-year travel time model. The completeness of the infrastructure was insignificantly associated with modal shift in most of the one-year models, but these associations turned significant in many of the two-year models.

## 4 Discussion

To determine the effectiveness of new walking and cycling infrastructure in promoting a modal shift we devised exposure measures to reflect both the distance between the intervention site and respondents’ homes, and the use of the infrastructure. The distance serves as a proxy for the accessibility of and *potential* exposure to the infrastructure, whereas the use can be interpreted as representing *actual* exposure. Strong and positive coefficients for the *‘use’* variable in our models indicate that experience of using the infrastructure was indeed positively associated with a modal shift from the private car towards walking and cycling. This is in line with the findings of Panter and Ogilvie (2015) that use of the new routes was the most important factor mediating the relationship between proximity to the infrastructure and physical activity behaviour change. On the other hand, distance from the intervention did not directly and independently predict modal shift, suggesting that passive or potential exposure to the new infrastructure may not have been sufficient to cause a modal shift as claimed by Jones (2012). However, it should be noted that walking and cycling infrastructure may be more often used for recreational activities, and that people often start cycling for leisure before they start cycling for transport (Jones, 2012, Smith et al., 2011). Connect2 users were also more likely to report using the infrastructure for recreational purposes than for utility journeys (Goodman et al., 2013, Goodman et al., 2014, Sahlqvist et al., 2015). Taken together, these findings suggest that new infrastructure of this kind could lead to an increase in walking and cycling in the general population in the medium term, initially mainly through recreational use (Cope et al., 2003) and later with additional use for more utility purposes.

**Fig. 4:**
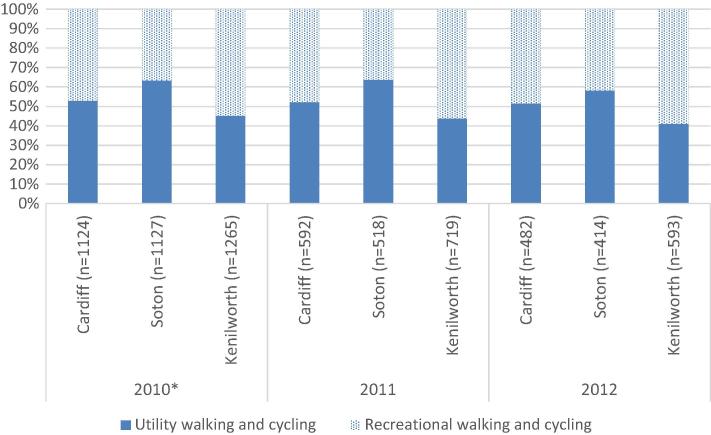
Proportions of time spent walking and cycling^∗∗^ reported for utility and recreational purposes by study site and year. ^∗^ All respondents’ data were used in calculations for 2010, applying a calibration weight based on local age and sex distribution similar to that previously described for the 2011 and 2012 data. ^∗∗^ Proportion of recreational (or utility) walking and cycling = $\frac{\text{average weekly time spent in recreational}(\text{or utility})\text{walking and cycling}}{\text{average weekly time spent in recreational and utility walking and cycling}}\times 100$.

The aggregate travel data showed regional variation in behaviour change. Southampton respondents were most likely to show the intended pattern of behaviour change, whilst those in Cardiff tended to reduce their active travel mode share. This somewhat contradicts the findings from Sahlqvist et al. (2015), a mixed method analysis of other data pertaining to the same study sites and samples, which found that people in Cardiff appeared the most supportive of the new infrastructure and the most likely to report using it. The apparent discrepancy reflects the different scopes of the two analyses. This paper concerns only walking and cycling for transport, whereas Sahlqvist et al. (2015) included both utility and recreational walking and cycling. The relative proportions of these differed by study site: people in Southampton tended to report spending more time walking and cycling for utility purposes than for recreation, with those in Kenilworth showing the opposite pattern and those in Cardiff reporting similar average times for both (Fig. 4). Such variation may be explained by the local contexts (Song et al., 2013, Sahlqvist et al., 2015) and completeness of the intervention at each site. The landmark People’s Bridge in Cardiff was constructed in 2011 and enabled a new recreational circuit for cycling around Cardiff Bay, but may have provided less immediate improvement for utility journeys especially without a satisfactory network of feeder routes. As presented in Appendix 1, infrastructure development in Cardiff was fragmented and not all surrounding areas had connecting walking and cycling routes when the follow-up surveys were conducted. Similar issues of incomplete and fragmented infrastructure development occurred in Kenilworth. On the other hand, the intervention in Southampton provided longer and more continuous routes for walking and cycling with linking routes already in place, providing better connectivity for utility travel. The positive and significant coefficients for the completeness variable in our models support this inference.

**Fig. 5:**
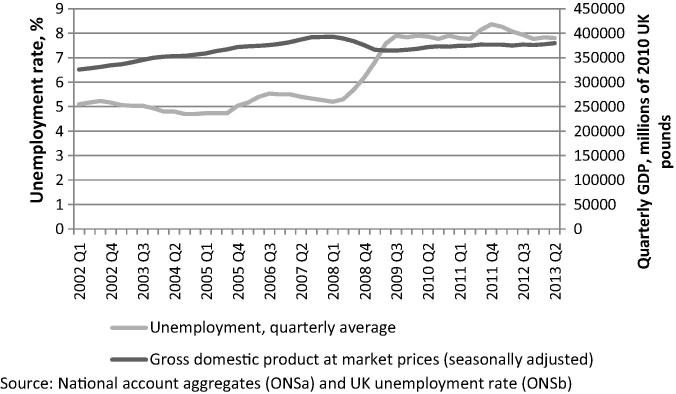
UK unemployment rate and GDP.

Our sample’s aggregate travel data show that respondents tended to travel less, and to choose less expensive modes of transport, in 2011 and 2012 than they did in 2010. This may reflect external factors at the time such as the economic downturn and rising fuel costs, as well as the ageing of the sample. The surveys were conducted whilst the UK economy was in the process of recovering from the recession provoked by the financial crisis in 2008. As shown in Fig. 5, gross domestic product (GDP) was slowly improving over the study period; however, unemployment was fluctuating around 8%, which was the highest level in the last 25 years (ONS, 2013a, ONS, 2013b). The respondents’ characteristics reported in Table 1 reflect the economic environment of the time, with decreases in the average number of household cars and the proportion of paid workers during the study. Their income also fell. Excluding those who did not respond to the income question, households earning over ₤50,000 a year accounted for 26% of respondents in 2010, but only 23.4% and 22.6% respectively in the following years. On the other hand, the proportion of households earning less than ₤20,000 a year increased from 30.9% in 2010 to 32.5% and 32.9% in 2011 and 2012 respectively^5^. Moreover, motor fuel prices increased by 13.5% and 15.4%^6^ over the one- and two-year periods respectively (DECC, 2013). Such economic conditions could have influenced personal travel behaviour through people trying to reduce their expenditure in the face of more insecure economic circumstances (Bernanke, 1981, Lucas, 1976) and the rising cost of using motor vehicles. This could be tested formally if a longer time series were available.

Several other factors were found to have significant associations with modal shift, of which two are emphasised here. First, change in employment status was found to be significant in many of the models, indicating that this might be an effective and efficient point at which to intervene and promote more sustainable travel behaviours by breaking the habitual use of private cars (Bamberg, 2006). It is an important life change at which people often change their travel behaviour to adapt to their new circumstances, and people who experience a significant life change are more likely to respond to changes in the relative attractiveness of different modes of transport (Fujii and Gärling, 2008, Santos et al., 2010). Those who have lost their job or retired are no longer required to commute at particular times and may have more free time to travel for other purposes. They may therefore be more willing to make journeys that take more time but do not necessarily cover a longer distance, as reflected in the results of the analysis, and could thereby be encouraged to integrate more active travel into their regular journeys and form new habits (Jones and Ogilvie, 2012, Verplanken et al., 2008). Second, more highly educated people were more likely to show a modal shift in our analysis. People with a higher level of education tend to have higher incomes and to travel more by motor vehicle, and therefore to contribute more to CO_2_ emissions (Brand et al., 2013, Thornton et al., 2011), and our sample consistently showed the same pattern in that those with a higher level of education travelled more, especially in terms of distance^7^. Although previous research has found that more highly educated people tended to have more pro-environmental attitudes but less sustainable travel behaviour (Anable et al., 2006, Thornton et al., 2011), the significant positive association between higher education attainment and modal shift in our analysis suggests that ‘high emitters’ may have been starting to move away from car use towards walking and cycling, even if only to a modest extent not yet sufficient to produce a significant overall effect on CO_2_ emissions (Brand et al., 2014). In addition, gender and ethnicity were found to be significant in many models, with females and ethnic minorities less likely to change their travel modes from car driving to active modes. This finding is in line with previous studies (Buehler et al., 2011, Merom et al., 2010, Steinbach et al., 2011).

This study used a postal survey to collect data on individual travel behaviour data as well as other individual and household characteristics. Data obtained in this way have well-known limitations (Dilman, 1978). For example, respondents could have wrongly interpreted the questions or intentionally or unintentionally tried to satisfy the researchers. However, the questionnaire was designed to minimise this risk (for example, by not indicating at baseline which intervention was the subject of the study, and by presenting the questions in exactly the same way to all respondents irrespective of their degree of exposure to the intervention) and we believe the risk of bias was reduced by using panel data^8^. Also, the calibration weights were constructed to reflect local population characteristics, not the whole country, and the findings of the study may therefore not necessarily be generalisable from our case study sites to other parts of the country or beyond.

## 5 Conclusions

Various strategies have been used to promote a shift from car use to more sustainable modes of transport in many countries (Graham-Rowe et al., 2011). In this study we aimed to evaluate the modal shift stimulated by improving infrastructure for walking and cycling. Using longitudinal panel data, we found that actual exposure to (use of) the infrastructure was significantly associated with a modal shift towards active travel after controlling for personal and household characteristics, but that passive exposure (residential proximity to the infrastructure) was not directly associated with a modal shift. Pucher and Buehler (2008) have argued that the provision of dedicated cycling facilities is critical for achieving a higher level of cycling, based upon aggregate international data. Our study suggests that while infrastructure provision may not be a sufficient condition to achieve this, it may well be a necessary condition in that the people who shifted towards more active travel tended to be those who were using the new infrastructure.

Behaviour change may take more than a year or two. Once people are habituated to using private motor vehicles, it may not be easy for them to change to modes of transport that are perceived as less comfortable or convenient and take more time to cover the same distance (Gärling and Axhausen, 2003, Thøgersen and Møller, 2008), unless there is a shock that significantly interrupts the habitual behaviour. Moreover, the planned infrastructure programmes were not fully implemented in two of our three case-study sites when follow-up surveys were conducted. Once these are completely in place, connecting communities as envisaged and enabling residents to more fully appreciate their potential, more enduring effects may emerge over time.

## Figures and Tables

**Figure:**
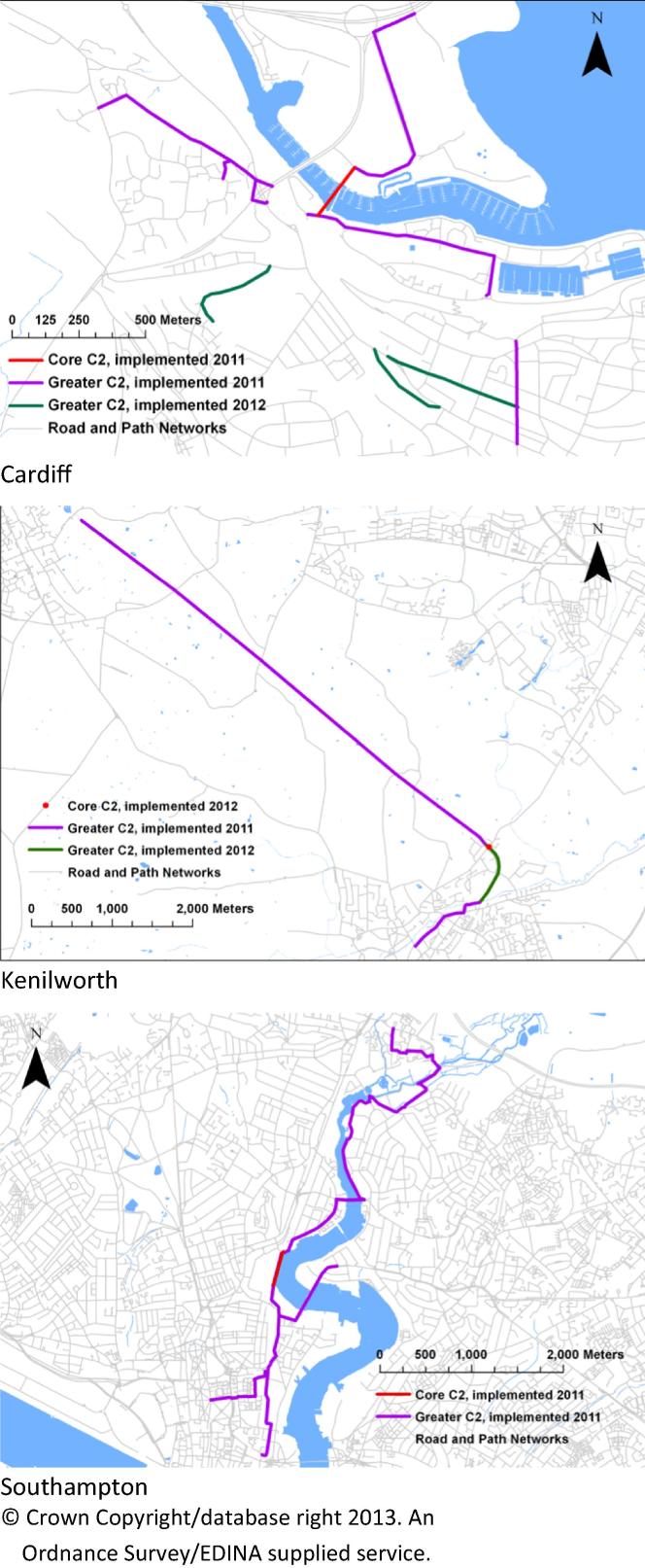


**Table 1:** Sample characteristics of all survey respondents.⁎

	2010 sample (n_1_ = 3516)	2011 sample (n_2_ = 1906)	2012 sample (n_3_ = 1564)
	Count (%)	Count (%)	Count (%)
Gender	n_1_ = 3496	n_2_ = 1906	n_3_ = 1564

Male	1577 (45.1)	880 (46.2)	688 (44.0)
Female	1919 (54.9)	1026 (53.8)	876 (56.0)

Education	n_1_ = 3424	n_2_ = 1866	n_3_ = 1484

University degree or higher	1377 (40.2)	751 (40.2)	610 (41.1)
Other	2047 (59.8)	1115 (59.8)	874 (58.9)

Ethnicity	n_1_ = 3463	n_2_ = 1895	n_3_ = 1559

White	3274 (94.5)	1821 (96.1)	1501 (96.3)
Other ethnic groups	189 (5.5)	74 (3.9)	58 (3.7)

Household cars/vans	n_1_ = 3195	n_2_ = 1795	n_3_ = 1323

No car	454 (14.2)	222 (12.4)	184 (13.9)
1 car	1273 (39.8)	785 (43.7)	574 (43.4)
2 or more cars	1468 (45.9)	788 (43.9)	565 (42.7)
Mean	1.45 per household	1.43 per household	1.40 per household

Work	n_1_ = 3470	n_2_ = 1899	n_3_ = 1528

Paid worker	1868 (53.8)	933 (49.1)	689 (45.1)
Student	223 (6.4)	43 (2.3)	24 (1.6)
Not working or studying	1379 (39.7)	923 (48.6)	815 (53.3)

Age⁎⁎	n_1_ = 3463	n_2_ = 1901	n_3_ = 1560

18–35	836 (24.1)	275 (14.5)	176 (11.3)
36–50	818 (23.6)	402 (21.1)	326 (20.9)
51–65	1030 (29.7)	662 (34.8)	573 (36.7)
66 and over	779 (22.5)	562 (29.6)	485 (31.1)
Mean (yrs)	50.9	55.7	57.1

⁎ *Note*: sample size for each variable and response combination varies due to non-response to each question.

⁎⁎ Age in 2010 was used for comparison.

**Table 2:** Mean baseline and within-participant changes in travel time and distance by mode.

	Baseline 2010–2011 (*n* = 1829)⁎		Change 2010–2011 (*n* = 1829)		Change 2010–2012 (*n* = 1489)	
	Time (min)	Distance (miles)	Time (min)	Distance (miles)	Time (min)	Distance (miles)
Walk	133.79	7.10	−7.56 (.104)	−0.27 (.307)	−13.30 (.021)	−0.15 (.573)
Cycle	23.22	3.81	−1.80 (.329)	−0.21 (.533)	+0.43 (.776)	+0.25 (.262)
Bus	37.65	6.85	−8.12 (.005)	−2.30 (.001)	−6.11 (.018)	+0.062 (.945)
Train	34.22	21.74	−8.75 (.004)	−1.67 (.470)	−7.67 (.046)	+5.03 (.045)
Car driver	238.65	101.87	−3.44(.611)	−4.12 (.243)	−14.54 (.055)	−10.71 (.007)
Car passenger	53.99	22.09	+0.96 (.800)	+2.08 (.335)	+13.29 (.001)	+8.02(.000)
Other	26.95	18.91	−1.96(.658)	+2.69 (.656)	−2.44 (.582)	−31.72(.009)

Total	548.47	182.38	−30.68 (.008)	−3.79 (.625)	−30.34 (.015)	−29.20 (.025)

Mean (p–value from two-tailed paired *t*-test).

⁎ Baseline travel time and distance by mode were measured from the 2010–2011 panel data. The baseline values are not exactly the same for the 2010–2012 panel due to different samples attained.

**Table 3:** Mean changes⁎ in modal splits for active travel and car driving by study site.

		2010–2011				2010–2012			
		Cardiff	Soton⁎⁎	Kenilworth	All	Cardiff	Soton	Kenilworth	All
	*n*	592	518	719	1829	482	414	593	1489
Active travel	Time	−1.18 (−4.2%)	+2.93 (+7.4%)	+0.91 (+3.4%)	+0.81 (+2.4%)	−2.26 (−7.9%)	+2.97 (+7.8%)	+0.98 (+4.9%)	+0.48 (+1.8%)
	Distance	−0.67 (−4.3%)	+3.57 (+14.7%)	+1.00 (+7.5%)	+1.19 (+6.4%)	−0.09 (−0.5%)	+1.78 (+7.4%)	+1.15 (+13.2%)	+0.92 (+6.3%)

Car driving	Time	+2.11 (+4.8%)	−2.02 (−5.5%)	−0.88 (−1.5%)	−0.24 (−0.3%)	+0.29 (+0.7%)	−2.76 (−8.3%)	−0.10 (−0.1%)	−0.71 (−1.5%)
	Distance	+1.42 (+3.0%)	−3.12 (−7.0%)	−1.36 (−2.2%)	−0.96 (−1.7%)	−3.12 (−5.5%)	−2.63 (−6.7%)	+0.32 (+0.6%)	−1.61 (−3.0%)

Proportionate changes are shown in parentheses, which were calculated for each individual and then averaged. Individual changes were calculated as $\frac{(\mathrm{Modal}\;\mathrm{split}\;\mathrm{in}\;2011\;\mathrm{or}\;2012-\mathrm{Modal}\;\mathrm{split}\;\mathrm{in}\;2010)\times 100}{\mathrm{Modalsplitin}2010}$.

⁎ Modal split (%) in 2011 or 2012 – modal split (%) in 2010.

⁎⁎ Soton: Southampton.

**Table 4:** Explanatory variables.

Type	Variables
Personal characteristics	Age, Female^d^, Ethnic minority^d^, University degree or higher^d^
Changes in socioeconomic status⁎	Job loss^d^, New job^d^, Δ Family size
Changes in access to private vehicles⁎	Δ Number of adult bikes in the household, Δ Number of cars and vans available to the household
Exposure to the new walking and cycling infrastructure⁎⁎	Use of the intervention^d^, Distance to Core C2, Distance to Greater C2
Completeness of infrastructure⁎⁎⁎	Percentage of infrastructure constructed by site

d Dummy variable, Δ Change in.

⁎ Change from the status or value in the 2010 baseline survey in the follow-up survey in 2011 or 2012.

⁎⁎ Distances in metres were computed using actual road and path networks in 2011.

⁎⁎⁎ Proportion of the planned total route length actually completed, calculated as $\frac{\mathrm{Total}\;\mathrm{route}\;\mathrm{length}\;\mathrm{actually}\;\mathrm{constructed}}{\mathrm{Total}\;\mathrm{route}\;\mathrm{length}\;\mathrm{planned}\;\mathrm{for}\;\mathrm{construction}}$.

**Table 5:** Associations with modal shift after one year.

Outcome modelled		Modal shift in terms of time share			Modal shift in terms of distance share		
Exposure modelled		C2 Use	Dist.⁎⁎ to Core C2	Dist. to Greater C2	C2 Use	Dist. to Core C2	Dist. to Greater C2
Change in socioeconomic status	Job loss	.96 (3.06)^‡^	.94 (2.98)^‡^	.93 (2.97)^‡^	.69 (2.03)^†^	.68 (1.99)^†^	.67 (1.98)^†^
	New job	.08 (.21)	.07 (.17)	.06 (.16)	.19 (.49)	.19 (.49)	.19 (.49)
	Change in family size	.16 (1.71)⁎	.16 (1.79)⁎	.16 (1.81)⁎	−01 (−0.05)	6.9 × 10^−5^ (.00)	1.5 × 10^−3^ (.01)

Change in access to private vehicles	Bike	.01 (.05)	.03 (.18)	.02 (.18)	−0.03 (−0.21)	−0.01 (−0.07)	−0.01 (−0.06)
	Cars	.15 (.89)	.16 (1.00)	.17 (1.02)	.08 (.53)	.09 (.55)	.09 (.54)

Personal characteristics	Age	3.7 × 10^−4^ (.08)	3.4 × 10^−4^ (.07)	3.1 × 10^−4^ (.07)	−4.7 × 10^−4^ (−0.10)	−3.4 × 10^−4^ (−0.07)	−3.4 × 10^−4^ (−0.07)
	Female	−0.32 (−2.07)^†^	−0.36 (−2.35)^†^	−0.36 (−2.32)^†^	−0.33 (−2.06)^†^	−0.37 (−2.30)^†^	−0.36 (−2.26)^†^
	Ethnicity	−1.27 (−2.35)^†^	−1.14 (−2.29)^†^	−1.12 (−2.24)^†^	.09 (.17)	.11 (.21)	.14 (.28)
	Education	.33 (2.04)^†^	.38 (2.33)^†^	.39 (2.39)^†^	.33 (1.94)⁎	.36 (2.08)^†^	.36 (2.12)^†^

Exposure⁎⁎⁎		.55 (3.26)^‡^	−4.2 × 10^−5^ (−0.94)	−5.8 × 10^−5^ (−0.80)	.43 (2.34)^†^	−5.5 × 10^−5^ (−1.18)	−8.3 × 10^−5^ (−1.41)
Completeness	% complete by site	.01 (1.87)⁎	4.8 × 10^−3^ (1.04)	5.0 × 10^−3^ (1.06)	−2.2 × 10^−3^ (−0.36)	−5.2 × 10^−3^ (−0.97)	−5.1 × 10^−3^ (−0.95)
Constant		−1.75 (−3.08)^‡^	−1.03 (−1.92)⁎	−1.10 (−2.05)^†^	−0.88 (−1.23)	−0.29 (−0.45)	−0.34 (−0.53)
*F-*value		3.47^‡^	3.01^‡^	2.98^‡^	1.99^†^	1.74⁎	1.79^†^

Coefficients (*t*-values).

⁎ significant at the 10% level, ^†^ significant at the 5% level, and ^‡^ significant at the 99% level.

⁎⁎ Dist.: distance.

⁎⁎⁎ Exposure refers to the three alternative measures of exposure to the intervention used in the models, which appear at the top of each result column.

**Table 6:** Associations with modal shift after two years.

Outcome modelled		Modal shift in terms of time share			Modal shift in terms of distance share		
Exposure modelled		C2 Use	Dist. to Core C2	Dist. to Greater C2	C2 Use	Dist. to Core C2	Dist. to Greater C2
Change in socioeconomic status	Job loss	.99 (7.61)^‡^	1.04 (8.16)^‡^	1.04 (8.16)^‡^	.81 (6.02)^‡^	.87 (6.69)^‡^	.87 (6.79)^‡^
	New job	−0.04 (−0.16)	−0.10 (−0.49)	−0.10 (−0.48)	−0.14 (−0.67)	−0.20 (−0.99)	−0.21 (−1.01)
	Change in family size	−0.19 (−3.85)^‡^	−0.18 (−3.61)^‡^	−0.18 (−3.63)^‡^	−0.08 (−1.64)	−0.07 (−1.30)	−0.07 (−1.30)

Change in access to private vehicles	Bike	.01 (1.88)⁎	.01 (2.56)^†^	.01 (2.54)^†^	−8.9 × 10^−4^ (−0.31)	2.0 × 10^−4^ (.74)	2.1 × 10^−3^ (.75)
	Cars	.15 (2.19)^†^	.20 (2.80)^‡^	.20 (2.78)^‡^	.04 (1.48)	.08 (1.11)	.08 (1.08)

Personal characteristics	Age	2.9 × 10^−3^ (1.20)	3.5 × 10^−3^ (1.45)	3.4 × 10^−3^ (1.43)	−3.3 × 10^−3^ (−1.21)	−2.5 × 10^−3^ (−0.93)	−2.4 × 10^−3^ (−0.90)
	Female	−0.26 (−3.20)^‡^	−0.29 (−3.55)^‡^	−0.29 (−3.52)^‡^	−0.37 (−4.33)^‡^	−0.40 (−4.67)^‡^	−0.40 (−4.64)^‡^
	Ethnicity	−0.60 (−2.77)^‡^	−0.69 (−3.12)^‡^	−0.70 (−3.14)^‡^	−1.08 (−4.70)⁎	−1.18 (−5.15)^‡^	−1.17 (−5.14)^‡^
	Education	.69 (8.41)^‡^	.81 (9.69)^‡^	.82 (9.70)^‡^	.33 (3.67)^‡^	.48 (5.19)^‡^	.48 (5.19)^‡^

Exposure⁎⁎⁎		.69 (8.54)^‡^	−1.3 × 10^−5^ (−1.45)	−1.3 × 10^−5^ (−1.60)	.79 (8.84)^‡^	−2.5 × 10^−7^ (−0.05)	−1.1 × 10^−5^ (−1.35)
Completeness	% complete by site	.02 (4.43)^‡^	.01 (2.16)^†^	.01 (2.20)^†^	.02 (2.94)^‡^	3.0 × 10^−3^ (.55)	2.9 × 10^−3^ (.52)
Constant		−4.01 (−8.40)^‡^	−2.61 (−5.62)^‡^	−2.65 (−5.74)^‡^	−2.83 (−5.37)^‡^	−1.37 (−2.79)^‡^	−1.34 (−2.73)^‡^
*F-*value		27.21^‡^	22.38^‡^	22.32^‡^	19.60^‡^	14.81^‡^	14.92^‡^

Coefficients (*t*-values).

⁎ Significant at the 10% level, ^†^ significant at the 5% level, and ^‡^ significant at the 1% level.

⁎⁎⁎ Exposure refers to the three alternative measures of exposure to the intervention used in the models, which appear at the top of each result column.

## Footnotes

1 More detailed information on the content and conduct of the survey as well as the full questionnaire can be found in Ogilvie et al. (2012).

2 During the same period the average number of household cars decreased from 1.17 to 1.15 and the proportion of households with access to multiple cars decreased by 2% in England (DfT, 2015).

3 The discrete choice model was chosen mainly for two reasons: (1) both aspects of travel behaviour change, i.e. towards active travel and away from car driving, can be simultaneously and effectively dealt with; and (2) continuous dependent variables such as level or share of active travel did not show clear associations with the relevant explanatory variables.

4 Each variable’s statistical significance can be assessed by the corresponding t-value which indicates whether the estimated coefficient is significantly different from 0 or not. The statistical significance levels are indicated with marks at three different levels (10%, 5% and 1%) in Table 5, Table 6.

5 Income level was not included in the statistical analysis because it was strongly correlated with the variable representing change in working status, and including it would have violated the assumption of non-collinearity.

6 Figures are based on ‘super unleaded’ motor spirit. Other types of motoring fuels underwent larger increases in price during the same periods, with the price of premium unleaded petrol rising by 14% and 15.8% and that of diesel rising by 16.3% and 18.9% (DECC, 2013).

7 In our sample, those with degree-level education or higher travelled 40–52% more in terms of distance and 1–20% more in terms of time.

8 Response reliability was tested before the baseline survey was carried out. This reliability study showed strong overall reliability for the whole questionnaire and for the record of travel behaviour (Adams et al., 2014). This suggests that the use of panel data, collected from the same group of people over time, would have reduced the impact of measurement error or bias in responses.
